# The Original Histological Slides of Camillo Golgi and His Discoveries on Neuronal Structure

**DOI:** 10.3389/fnana.2019.00003

**Published:** 2019-02-18

**Authors:** Marina Bentivoglio, Tiziana Cotrufo, Sergio Ferrari, Chiara Tesoriero, Sara Mariotto, Giuseppe Bertini, Antonella Berzero, Paolo Mazzarello

**Affiliations:** ^1^Department of Neuroscience, Biomedicine, and Movement Sciences, University of Verona, Verona, Italy; ^2^National Institute of Neuroscience (INN), Verona, Italy; ^3^Golgi Museum, University Museum System of Pavia, Pavia, Italy; ^4^Department of Brain and Behavioral Sciences, University of Pavia, Pavia, Italy

**Keywords:** Golgi staining, axon collaterals, cerebral cortex, hippocampus, dendritic spines, Purkinje cells, history of neuroscience

## Abstract

The metallic impregnation invented by Camillo Golgi in 1873 has allowed the visualization of individual neurons in their entirety, leading to a breakthrough in the knowledge on the structure of the nervous system. Professor of Histology and of General Pathology, Golgi worked for decades at the University of Pavia, leading a very active laboratory. Unfortunately, most of Golgi's histological preparations are lost. The present contribution provides an account of the original slides on the nervous system from Golgi's laboratory available nowadays at the Golgi Museum and Historical Museum of the University of Pavia. Knowledge on the organization of the nervous tissue at the time of Golgi's observations is recalled. Notes on the equipment of Golgi's laboratory and the methodology Golgi used for his preparations are presented. Images of neurons from his slides (mostly from hippocampus, neocortex and cerebellum) are here shown for the first time together with some of Golgi's drawings. The sections are stained with the Golgi impregnation and Cajal stain. Golgi-impregnated sections are very thick (some more than 150 μm) and require continuous focusing during the microscopic observation. Heterogeneity of neuronal size and shape, free endings of distal dendritic arborizations, axonal branching stand out at the microscopic observation of Golgi-impregnated sections and in Golgi's drawings, and were novel findings at his time. Golgi also pointed out that the axon only originates from cell bodies, representing a constant and distinctive feature of nerve cells which distinguishes them from glia, and subserving transmission at a distance. Dendritic spines can be seen in some cortical neurons, although Golgi, possibly worried about artifacts, did not identify them. The puzzling intricacy of fully impregnated nervous tissue components offered to the first microscopic observations still elicit nowadays the emotion Golgi must have felt looking at his slides.

## Introduction

The contributions of Camillo Golgi (1843–1926) to the study of the nervous system are a pillar of modern neuroscience. The Golgi impregnation first offered to microscopic studies individual neurons and glial cells in their entirety, and has therefore laid the foundation of neurohistology and neuroanatomy, opening a new era in neuroscience. Golgi's “black reaction” (*reazione nera*) was based on the fixation of nervous tissue blocks in potassium dichromate (2–2.5%) for a variable number of days or weeks (from 1 to 50 days or even longer), followed by immersion in silver nitrate which led to the precipitation of silver chromate fully impregnating cells in the nervous tissue (Golgi, 1873, 1885).

Golgi himself engaged for 10 years in seminal investigations on different brain structures, published in a monograph on the “fine anatomy of the central organs of the nervous system” which appeared in 1885 (Golgi, 1885), was republished the following year (Golgi, 1886) and is also part of his Opera Omnia (Golgi, 1903). The Golgi impregnation provided a revolutionary tool to the studies of other investigators and in particular to the monumental work of Santiago Ramón y Cajal (1852–1934) on the structure of the nervous system (Cajal, 1909). Cajal saw for the first time Golgi-impregnated sections in 1887, 14 years after Golgi's publication of the method. He stated in his autobiography that the method was then “unknown to the immense majority of neurologists or was undervalued” (Cajal, 1989) and described as a lightning in his life the observation of the impregnated elements as “drawings with Indian ink” (Cajal, 1909, 1989).

The scientific debate between Golgi, who defended the reticular theory of nerve cells continuity, and Cajal, paladin of the neuron doctrine, is at the roots of neuroscience (Shepherd, 2016; Mazzarello, 2018). After years of debates, the neuron doctrine, which stated that neurons are individual elements representing the structural and functional units of the nervous system, became the fundamental paradigm.

A historical re-evaluation of the original histological preparations of these two founders of modern neuroscience has been carried out, so far, only for Cajal. He has left to posterity about 1,500 slides of the nervous system, about 800 of which are impregnated by the Golgi method (Garcia-Lopez et al., 2010). Unfortunately, in the decades following Golgi's death, most of his slides have not been fully preserved in the Institute of General Pathology of the University of Pavia, where Golgi's laboratory had been located, and are lost.

We here present images of neurons from original slides on the nervous system of Golgi's laboratory currently available at the University of Pavia, with notes on his laboratory equipment and methodology for the preparation of the slides. Some of Golgi's drawings are also presented to show how he depicted microscopic observations, and his novel findings on neuronal structure are summarized.

## Biographical Sketch of Camillo Golgi

A detailed account of Golgi's life and work is provided by the biography written by Mazzarello (2010). A synopsis is here presented to recall the chronology of Golgi's main contributions.

Golgi was born in 1843 in Corteno, a village in the Alps of Lombardy. He graduated in medicine in 1865 at the University of Pavia, and started his clinical activity at the San Matteo Hospital in Pavia. He soon became assistant of Cesare Lombroso (1835-1909), the psychiatrist who became the father of criminal anthropology. In the Psychiatric Clinic, Golgi developed a great interest on neuropsychiatric diseases and on the brain. When free from clinical duties he attended the Institute of General Pathology, directed by Giulio Bizzozero (1846–1901).

An exceptionally talented investigator, 3 years younger than Golgi, Bizzozero discovered in Pavia the erythropoietic function of bone marrow and the phenomenon of phagocytosis. Later on, while professor of General Pathology at the University of Turin, he demonstrated the existence of blood platelets and classified into “stable,” “labile,” and “everlasting” the tissues of the body, a dogma for cell biology for almost a century (Mazzarello et al., 2001). Bizzozero introduced Golgi to experimental science and microscopic investigations.

Between 1870 and 1872, Golgi published histological studies, in particular on neuroglia. Under financial pressure, Golgi took then the post of Chief Physician at the hospital for the chronically ill (*Pio Luogo degli Incurabili*) in Abbiategrasso, about 30 km from Pavia. He set up there, in the kitchen of his apartment, a rudimentary laboratory, where he worked out his “black reaction” in 1873. As demonstrated by his private correspondence and his previous scientific work, Golgi was searching for new stains to crack the code of the structure of the nervous system (Mazzarello, 2010). As explained below, his new method combined reagents he had already used.

Golgi returned to the University of Pavia in 1876 as professor of Histology. 2 years later he described two kinds of tendinous sensory corpuscles: the Golgi tendon organs (proprioceptors) and the Golgi-Mazzoni corpuscles (transductors of pressure stimuli). In 1879 Golgi was also appointed as professor of General Pathology, with clinical responsibilities of a medical ward at the San Matteo Hospital. He saw patients until the end of the First World War (though refusing a paid private practice) and was very interested in diseases, in particular in infectious diseases.

Golgi focused on studies on the nervous system until 1885. When, in 1887, Cajal (9 years younger than Golgi) saw slides stained by the “black reaction,” Golgi had switched to studies on human malaria he pursued until 1892. In these investigations he obtained seminal findings, describing the intra-erythrocytic cycle of malaria parasites (*Plasmodium*) responsible for tertian and quartan fever (Golgi cycle), discovering the relationship between the febrile bouts and the microbe segmentation (Golgi law) and defined the timing, during this cycle, for an effective treatment with quinine. Golgi also engaged in histological studies of various organs, e.g., the kidney and gastric glands, on which he made important observations.

Possibly stimulated by the success of Cajal and the neuron doctrine, Golgi resumed examination of the nervous tissue, modifying the recipe of his impregnation, probably to reduce the intensity of metallic precipitates and to obtain less darkly stained preparations. Serendipity then played a role, and in 1898 Golgi, using a rapid variant of his chromoargentic impregnation, reported his discovery, in neurons of spinal ganglia, of the “internal reticular apparatus” (Mazzarello and Bentivoglio, 1998), the cell organelle later called after him Golgi apparatus or simply “the Golgi” (Fabene and Bentivoglio, 1998; Mazzarello et al., 2009). He also described in the same year the “perineuronal nets,” now known as key components of the brain extracellular matrix (Celio et al., 1998; Spreafico et al., 1999).

**Figure 1:**
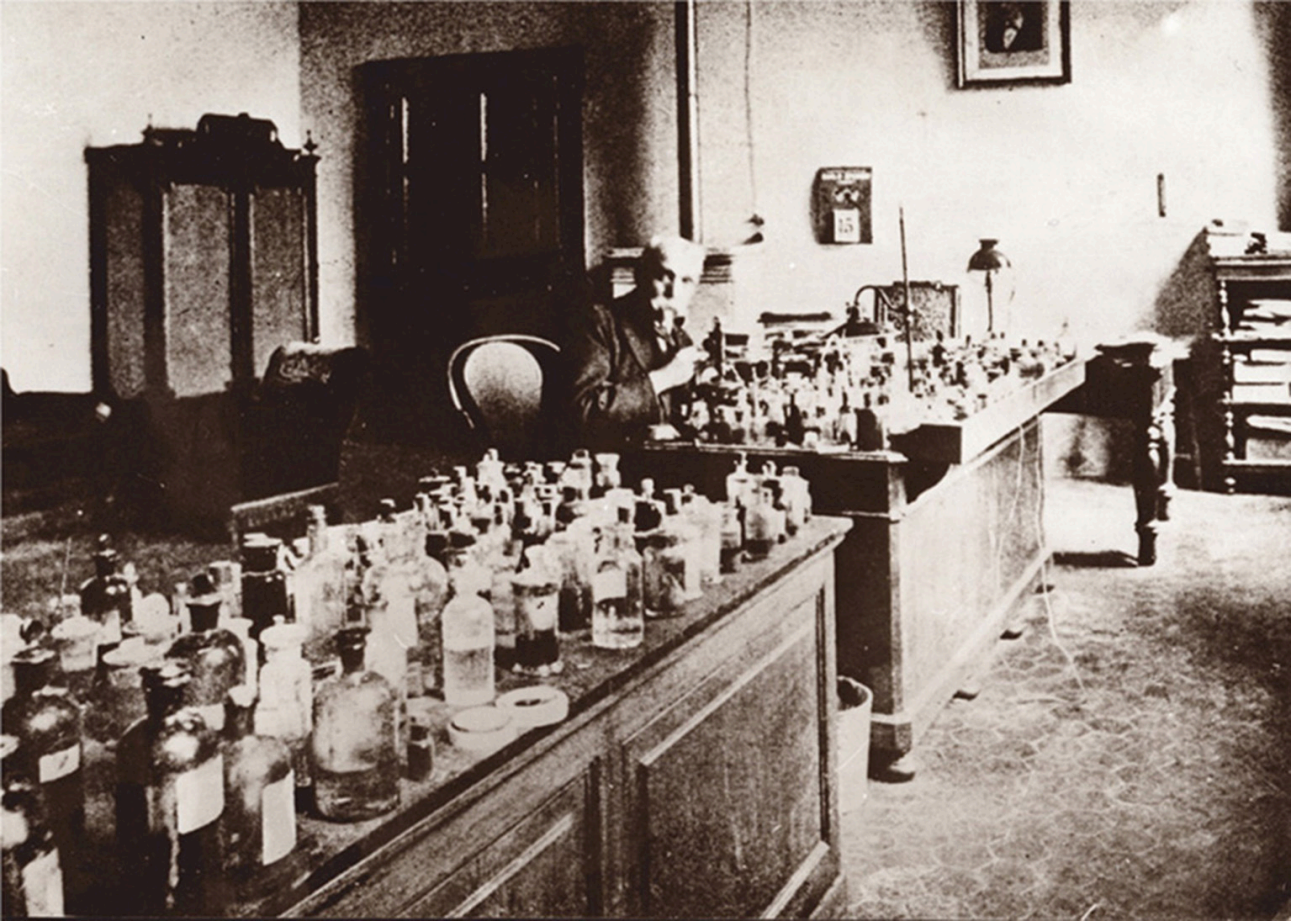
Camillo Golgi at his laboratory bench in the Institute of General Pathology of the University of Pavia around 1920. Reproduced with permission of the University Museum System of Pavia.

As it will be shown below, slides presented here are signed by Golgi with the indication of the year 1899, demonstrating that at the end of the century he was still examining brain tissue. On the other hand, a photograph of Golgi at his desk in the early 1920s shows that toward the end of his life he was still surrounded by little jars containing tissue blocks soaking in solutions (Figure 1).

Golgi led a very active laboratory at the Institute of General Pathology (Mazzarello, 2010, 2011), was Rector of the University of Pavia (1893–1896; 1901–1909) and became Senator of the Kingdom of Italy in 1900, focusing on problems of public health.

In 1906 Cajal and Golgi were awarded the Nobel Prize in Physiology or Medicine “in recognition of their work on the structure of the nervous system.” In their Nobel lectures, Golgi still stubbornly defended his early ideas supporting a reticular organization of the nervous system, while Cajal passionately supported the neuron doctrine. This event greatly damaged Golgi's reputation in neuroscience (Mazzarello, 2018), despite the importance of his observations, which will be here recalled.

## Notes on Knowledge on Nervous Tissue Structure at Golgi's Times

Theories and knowledge on the anatomical organization of the nervous system are here summarized to set the stage of the first observations made by Golgi with his method.

The cell theory, enunciated by Matthias Jakob Schleiden (1804–1881) and Theodor Schwann (1810–1882) in 1838–1839, stated that all animal and vegetal tissues and organs are formed by cells as structural and functional units. This theory, which was further developed by Robert Remak (1815–1865) and Rudolf Virchow (1821–1902) in the following 20 years, was not applied clearly to the nervous system to explain its fine structure.

**Figure 2:**
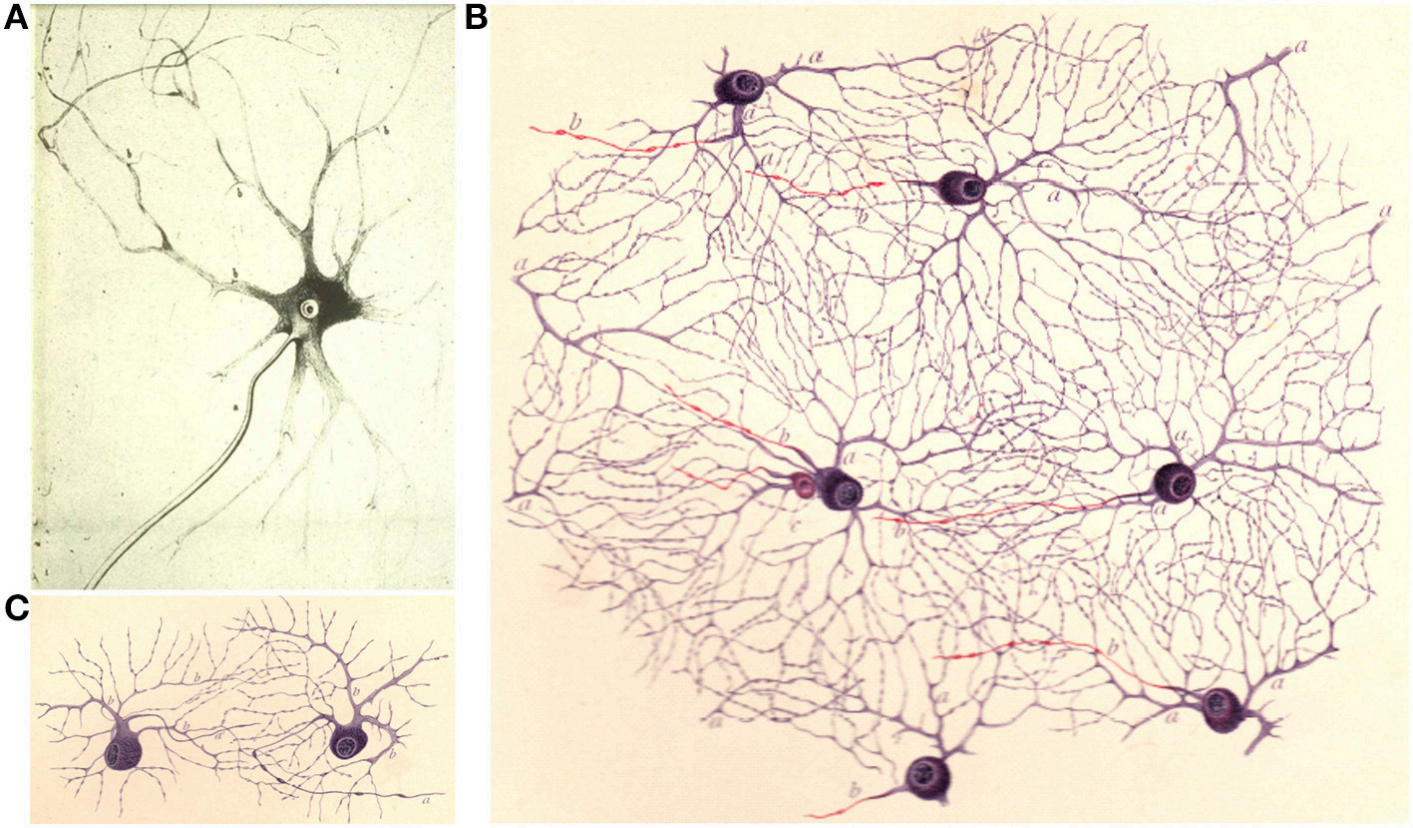
**(A)** Drawing from Deiters (1865) of an “isolated ganglion cell from the gray matter of the spinal cord… a, the main axis cylinder extension, b,b,b, the fine axis cylinder extensions coming from the protoplasmic extensions.” (translation in Shepherd, 2016). **(B,C)** Drawings by Dogiel (1891) illustrating a reticular dendritic interconnection (“protoplasmic net”). in the retina; the axons are in red.

When Golgi introduced the “black reaction,” the leading theory on the organization of the nervous system was the reticular theory, championed by the German anatomist Joseph von Gerlach (1820–1896). According to the theory postulated by Gerlach in 1871–72, the nervous system was a syncytium, a protoplasmic network formed by minute ramifications of nerve cell dendrites, giving origin also to nerve fibers (Figures 2B,C). The essential element of interconnection in the nervous tissue was therefore constituted by the richness of dendritic arborizations (Gerlach, 1872; Van Gehuchten, 1897; DeFelipe, 2010; Shepherd, 2016) (Figures 2B,C). Before Gerlach theory, Karl Deiters (1834–1863) had drawn neurons dissociated with a needle under the microscope from the ox spinal cord (Deiters, 1865), showing its processes, a work Golgi had much appreciated (Mazzarello, 2010). The axon was depicted by Deiters as a straight single process, unbranched in its initial segment emerging from the cell body, with additional fine processes originating from the dendrites (Figure 2A) (see also Deiters and Guillery, 2013; Shepherd, 2016).

The histological stains mostly used at that time were hematoxylin and carmine, and Gerlach himself had devised carmine staining modifications, which, however, resulted in a poor resolution of tissue components. The revolutionary Nissl stain, still in use nowadays as routine histological stain for nervous tissue cytoarchitecture, was introduced by Franz Nissl (1860–1919) about 10 years after the Golgi impregnation, in his MD thesis (Nissl, 1885). The Nissl stain, based on dyes (thionin or toluidine blue or cresyl violet) with affinity for basophilic cell components, allows a visualization of cell bodies and neuropil much clearer than carmine, but does not reveal the full extent of cell ramifications in the nervous tissue.

Histological techniques based on silver or silver/gold impregnation are different from classical stains. While the latter rely on the chemical affinity between a dye and a tissue or cell component (e.g., the alkaline cresyl violet binding to acidic RNA in the Nissl stain), metal impregnation results from the preferential precipitation of metal salts onto certain structures, for reasons that are not fully understood.

The crucial step in the development of Golgi's “black reaction” was the use of silver nitrate instead of hematoxylin or carmine after tissue fixation in potassium dichromate. Golgi had used potassium dichromate to harden the nervous tissue before sectioning in his studies on glia pursued, as mentioned above, in 1870–1872. He had also used potassium dichromate and silver nitrate to study the so-called “lymphatic” (perivascular) system of the brain (Golgi, 1870; Mazzarello, 2010). Silver nitrate was already in use in histology because it impregnates the intercellular substance in epithelia (including endothelia) and in connective tissues.

Concerning theoretical approaches, the neuron doctrine, enunciated in 1886–1887 by anatomists such as Wilhelm His (1831–1904), August Forel (1848–1931), Fridtjof Nansen (1861–1930) and boosted by Cajal's indefatigable work since 1888, was officially promulgated in 1891 by Wilhelm Waldeyer (1836–1921), who also christened the “neuron” (Bentivoglio and Mazzarello, 2010; Shepherd, 2016).

The term dendrite was proposed by Wilhelm His in 1889. Dendrites were called “protoplasmic processes” or “protoplasmic prolongations,” as also defined by Golgi in his “fine anatomy” (Golgi, 1885; see also Golgi et al., 2001). The axon was called by Golgi “nervous prolongation.” The term axon was officially introduced by Rudolph Albert von Kölliker (1817–1905) in 1896 (Bentivoglio, 1996). The term synapse was coined by Sir Charles Sherrington (1857–1952) in 1897 (Shepherd, 2016).

The concept of glia (*Nervenkitt*, “neuroglia”) had been introduced by Virchow in 1858 as “…that substance which lies between the proper nervous parts, holds them together…” (cited from: Kettenmann and Verkhratsky, 2008). The term astrocyte was proposed by Michael von Lehossék (1863–1937) in 1893 (Kettenmann and Verkhratsky, 2008).

At the turn of the 20th century, the nervous tissue was ready to surrender its secrets to new discoveries.

## Materials and Methods

### The Slides

**Figure 3:**
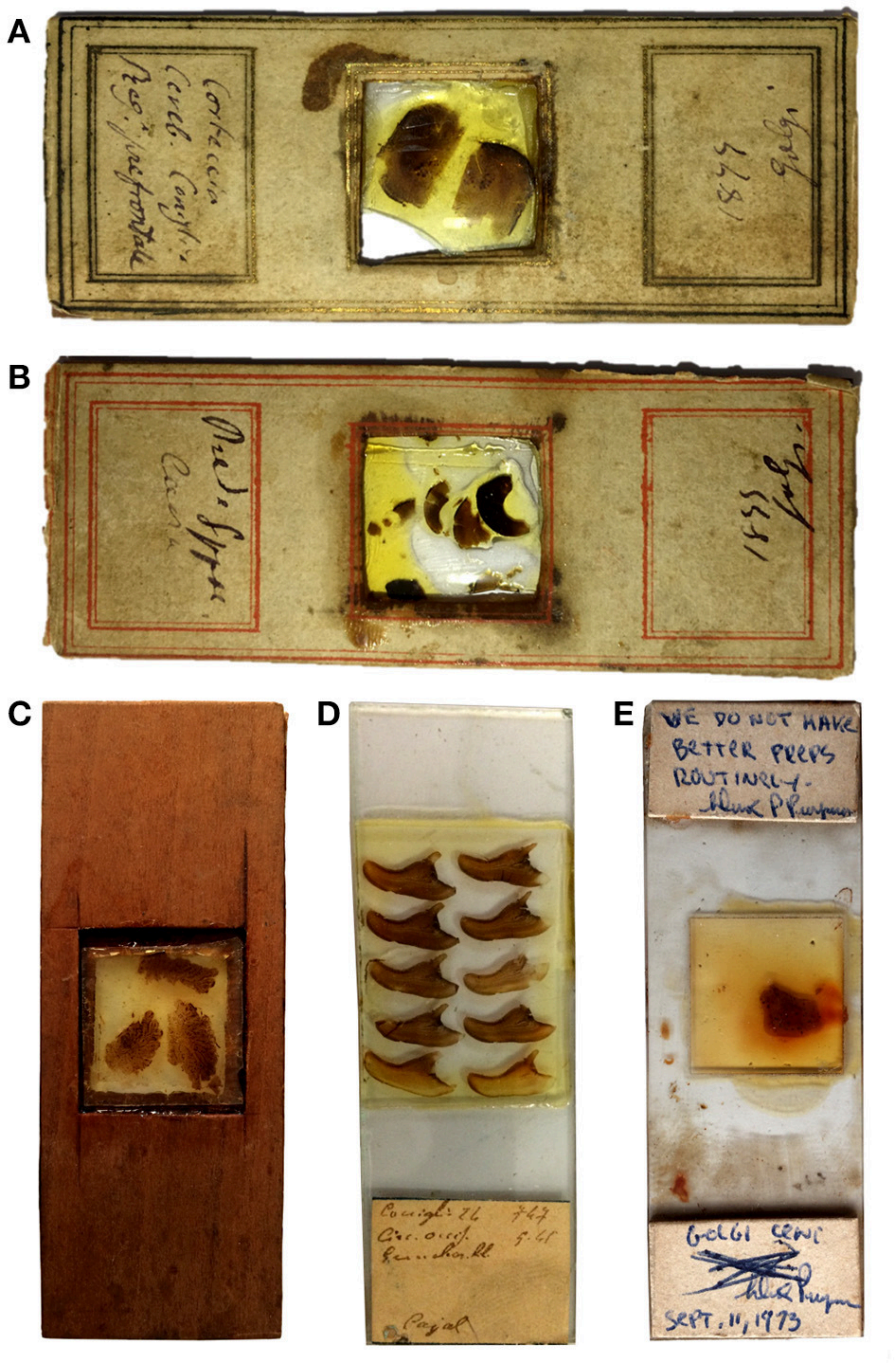
Photographs of some of the slides. The labels in **(A,B)** are signed by Golgi with the indication of the year 1899; **(C)** shows an example of a wooden slide; the label in **(D)** has the indication “Cajal”; the label in **(E)** has a comment signed by Dominick Purpura in 1973 (see text).

We examined 38 slides of nervous tissue from Golgi's laboratory (Table 1), currently kept at the Golgi Museum and at the Historical Museum in Pavia (which are part of the University Museum System). The labels of six slides are signed by Golgi, five with the indication of the year 1899 and one of the year 1877 (Figure 3). The label of one slide was signed by Dominick P. Purpura (Figure 3) at the time of the Golgi centennial symposium held in Pavia and Milan, September 9–12, 1973.

**Table 1:** Slides of nervous tissue from Golgi's laboratory available at the University of Pavia.

**Tissue**	**Number of slides**	**Label, slide material**
Cerebral cortex	9	3 Yes, wooden 1 No, wooden 4 Yes, glass 1 Yes, cardboard
Cerebellum	13	10 No, wooden 1 Yes, wooden 2 No, glass
Hippocampus	9	4 No, wooden 5 Yes, wooden
Dorsal root ganglia	2	1 No, wooden 1 Yes, glass
Spinal cord	4	3 No, wooden 1 without support
“Dissociated neurons”	1	1 Pen writing, glass

The slides are wooden, without coverslip, or glass slides with coverslips (Figure 3). The wooden slides contain thick sections covered by dammar resin and mounted on a coverslip nestled in a central window (Figure 3).

One of the coverslipped glass slides has a pen writing “*cellula nervosa unica*” (one nerve cell), and contains dissociated neurons.

### Notes on Golgi's Equipment

Golgi cut sections by hand with a razor or with a microtome, and used the camera lucida for his drawings (Mazzarello, 2010). Samples of the equipment used in Golgi's laboratory are on display at the Golgi Museum (Berzero et al., 2018).

**Figure 4:**
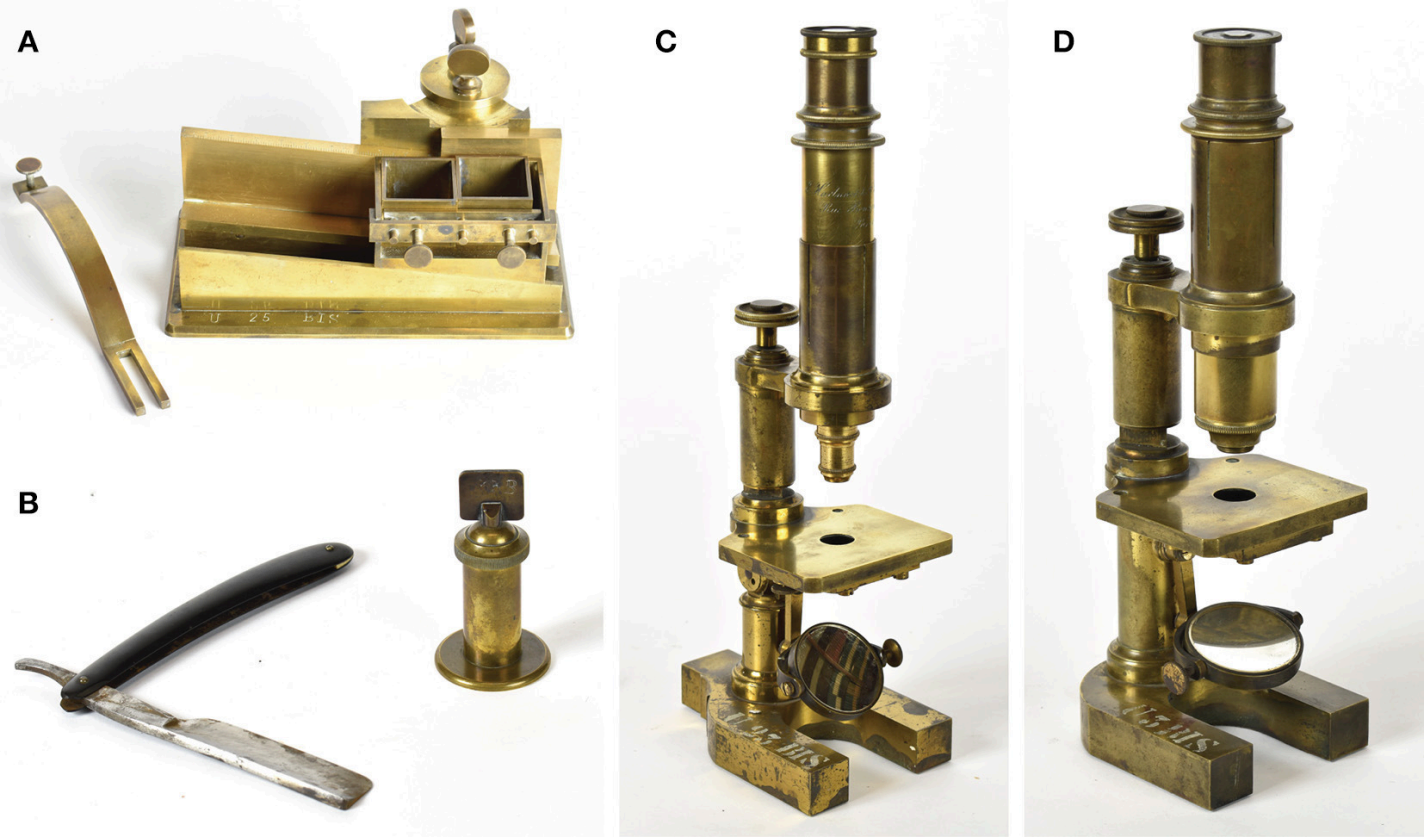
Equipment of the laboratory of Camillo Golgi in the years that followed his appointment as Professor of Histology at the University of Pavia in 1876, when Golgi's studies focused on the nervous system. The equipment is on display at the Golgi museum (Berzero et al., 2018). **(A)** Microtome by the German anatomist and physiologist Gustav Fritsch (1838–1927), bought in 1878. **(B)** Microtome by the French histologist and anatomist Louis Ranvier (1835–1922) to cut by hand, with the razor shown in the figure, sections sufficiently thin for microscopic examination from tissue blocks fixed to the cylinder; this microtome was bought in 1879. **(C)** Hartnack-Prazmowski microscope, bought in 1877 from the firm Hartnack had established in Paris in partnership with the Polish mathematician and astronomer Adam Prazmowski (1821–1885). **(D)** Microscope by Edmund Hartnack (1826–1891), renowned German microscope maker, bought in 1876.

Soon after Golgi's appointment as Professor of Histology in 1876, his laboratory acquired, in 1876 and in 1877, Hartnack microscopes, that were state of the art at the time (Figures 4C,D). Microtomes were bought in the following years: a Fritsch microtome in 1878 (Figure 4A), and a Ranvier microtome in 1879 (Figure 4B). A camera lucida Oberhauser was bought in 1877, a camera lucida Hartnack-Prazmowski in 1879, and a camera lucida Nachet in 1880. Besides the Hartnack microscopes, a Nachet microscope (1875), a Zeiss microscope (1880) a Koritska microscope (1887) were also part of Golgi's laboratory equipment.

Sliding microtomes (Becker microtome, Thoma-Jung microtome bought in 1885) followed, and a Becker freezing microtome was acquired in 1906. A Zeiss binocular eyepiece was probably acquired at the end of the Nineteenth century.

### Golgi's Notes on the Preparation of the Slides

In his “fine anatomy,” Golgi (1885) provided some methodological details of the preparation of the slides. First of all, he recommended to soak tissue blocks in absolute alcohol to clear silver nitrate impregnation. He also recommended to change the alcohol solution two, three or more times, until the tissue becomes transparent, specifying that “after about 9 years of storage of the specimens this way, I can obtain an impregnation as clear as with fresh pieces.”

Golgi also stated that, before treatment with Canada balsam or dammar resin, the section handling required two steps: (a) very careful washing two or three times in absolute alcohol, which ensures long-term preservation of the sections; (b) clearing (*rischiaramento*): “after absolute alcohol the sections should be soaked first in creosote for several minutes and then in turpentine oil, where they can be left for a long time.” Golgi emphasized that this protocol ensured an optimal preservation of the sections, that had then be covered by dammar resin (this being more satisfactory than Canada balsam).

Furthermore Golgi stated that, at variance with the usual procedure for microscopic preparations, the impregnated sections should not be coverslipped, since after coverslipping they become yellowish, the impregnated cell images become less distinct, the tissue becomes opaque and within a certain period of time (“about 2 or 3 years”) the sections become useless. “A thin layer” of dammar resin can instead preserve the quality of the sections for several years. If the sections start to deteriorate, their transparency can be recovered with a prolonged bath of the entire slide in turpentine oil.

“To preserve the sections I have found convenient to use wooden slides with a hollow square window where I apply, with a lacquer dissolved in alcohol, a glass coverslip” stated Golgi (Golgi, 1885), explaining that he used a coverslip as support for the section he then covered with dammar resin. He specified that this procedure “allows to examine the sections from both sides” and protects them from dust or external damage, especially when keeping the section facing downwards (and the supporting coverslip facing upwards) after solidification of the dammar resin.

Finally, Golgi recommended to keep the preparations in the dark avoiding exposure to light, though this was not an absolute requirement.

### Current Analyses

The slides presented here have been examined with an Olympus BX63 microscope equipped with a 40X objective (numerical aperture 0.65) under bright-field illumination. Objectives with a magnification higher than 40X and oil immersion objectives were not used to avoid damaging the slides. Images were taken with a QUICAM Fast 1394 digital camera.

For 3D reconstruction, images were acquired in grayscale using the Stereoinvestigator software and Neurolucida (v10.42.1, MBF, Inc.). This system was used to capture image stacks through the z-depth of the tissue at 0.3 μm steps. Image stacks were inverted and deconvolved by using the Huygens deconvolution algorithm (Scientific Volume Imaging BV, Hilversum, Netherlands) to decrease blurring and noise of the samples. Image stacks were finally imported into the IMARIS software (BitPlane, Zurich, Switzerland) for analysis and 3D reconstruction.

Section thickness was evaluated by measuring the distance along the z axis across the features of the specimen that could be placed in focus.

## Results: Microscopic Images

### Golgi Impregnation

As it is well-known, the Golgi impregnation fills at random a limited number of cells in the nervous tissue, which is the still unexplained secret of its revelatory power. The impregnated cells stand out in black over a light yellow background.

The sections contain the unavoidable precipitates of metallic impregnation, more abundant in some sections than in others, and in some parts of each section. Blood vessels are mostly heavily black-stained. In some fields, however, neurons and glia stand out very clearly, a view which comes almost as a surprise while screening the sections.

We here present images of neurons from slides of the hippocampus and neocortex, which are those of best quality, and comment also on slides containing cerebellar tissue. The observations of glial cells will be presented in a separate contribution.

The sections are very thick, and require continuous adjustments of the focal plane (Supplementary Movie 1). In one section of the hippocampus and one of the cerebral cortex we were able to evaluate a thickness between 150 and 200 μm.

**Figure 5:**
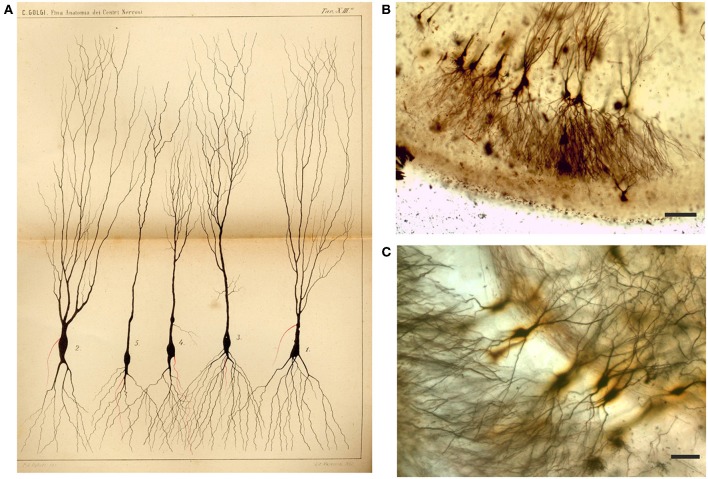
Drawing **(A)** and images **(B,C)** from Golgi-impregnated *pes Hippocampi major* (Ammon's horn) of the rabbit. **(A)** The drawing is Plate XIII from Golgi (1885), the translation of the original figure legend is provided by Bentivoglio and Swanson in Golgi et al. (2001). In the figure legend, Golgi noted the “different shapes presented by these cells.” The initial part of the “nerve process” (the axon) is drawn in red, and Golgi noted in the legend that “it should be considered a general rule that this part ramifies into numerous secondary fibrils that branch profusely.” **(B,C)** Impregnated neurons in Golgi's slides, showing what he should have seen. Scale bars: 200 μm in **(B)**, 50 μm in **(C)**.

**Figure 6:**
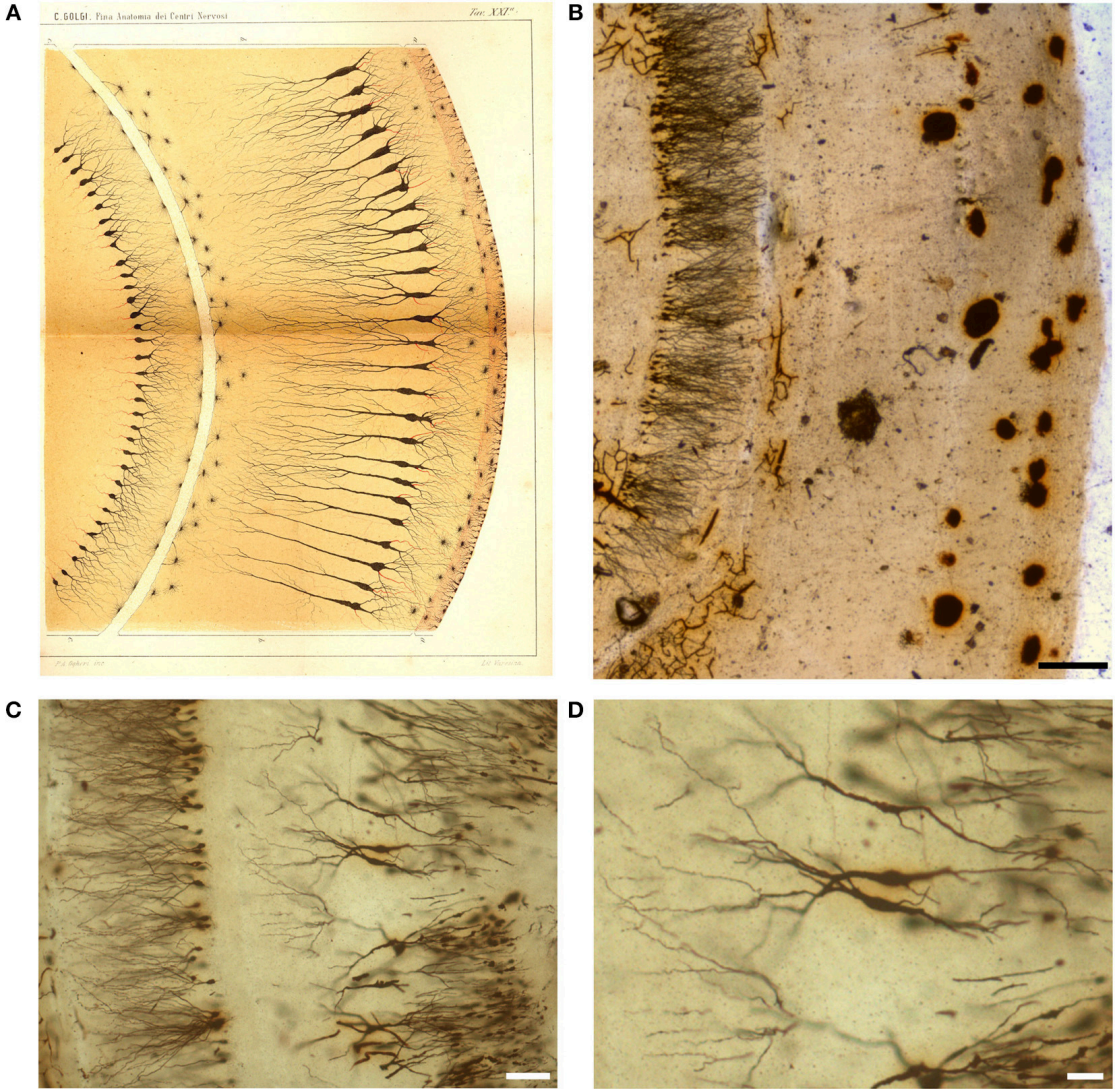
Drawing **(A)** and images **(B–D)** from a Golgi-impregnated “vertical section” through the *pes Hippocampi major* (Ammon's horn) of the rabbit. **(A)** The drawing is Plate XXI from Golgi (1885), and, as for Figure 5, the translation is provided by Bentivoglio and Swanson in Golgi et al. (2001). In the figure legend Golgi described a “ventricular epithelium,” composed by cells “strikingly analogous to. neuroglial cells,” a “convoluted gray layer,” and “small nerve cells of the fascia dentata.” **(B–D)** Images from a Golgi's slide. Scale bars: 200 μm in **(B)**, 30 μm in **(C)**, 50 μm in **(D)**.

**Figure 7:**
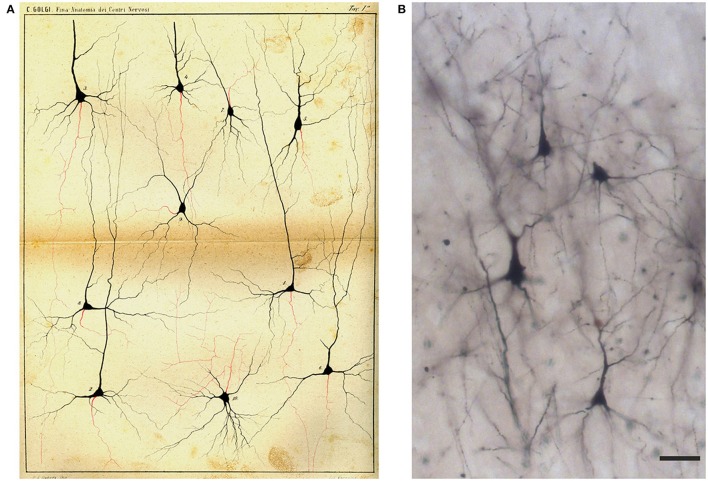
Drawing **(A)** and images **(B)** of Golgi-impregnated neurons of the cerebral cortex. **(A)** The drawing is Plate I from Golgi (1885). In the legend Golgi stated that the plate, illustrating “some types of ganglion cells in the cerebral cortex,” is especially destined to show the origin and branching of the axon (“the only nervous prolongation of each ganglion cell”); the cells are from the frontal (“anterior central”; cells 1, 2, 4, 5, 9, 10) and occipital (cells 3, 6–8) human cerebral cortex. The legend states that, on the basis of their axonal ramifications, cells 1 and 3 illustrate examples of the first type of neurons (currently named as “Golgi type I,” see text) and cell 2 an example of the second type (currently “Golgi type II”). The legend also states that the axon could not be followed because it became too thin “destined to get lost in the diffuse net.” Scale bar in **(B)**: 30 μm.

**Figure 8:**
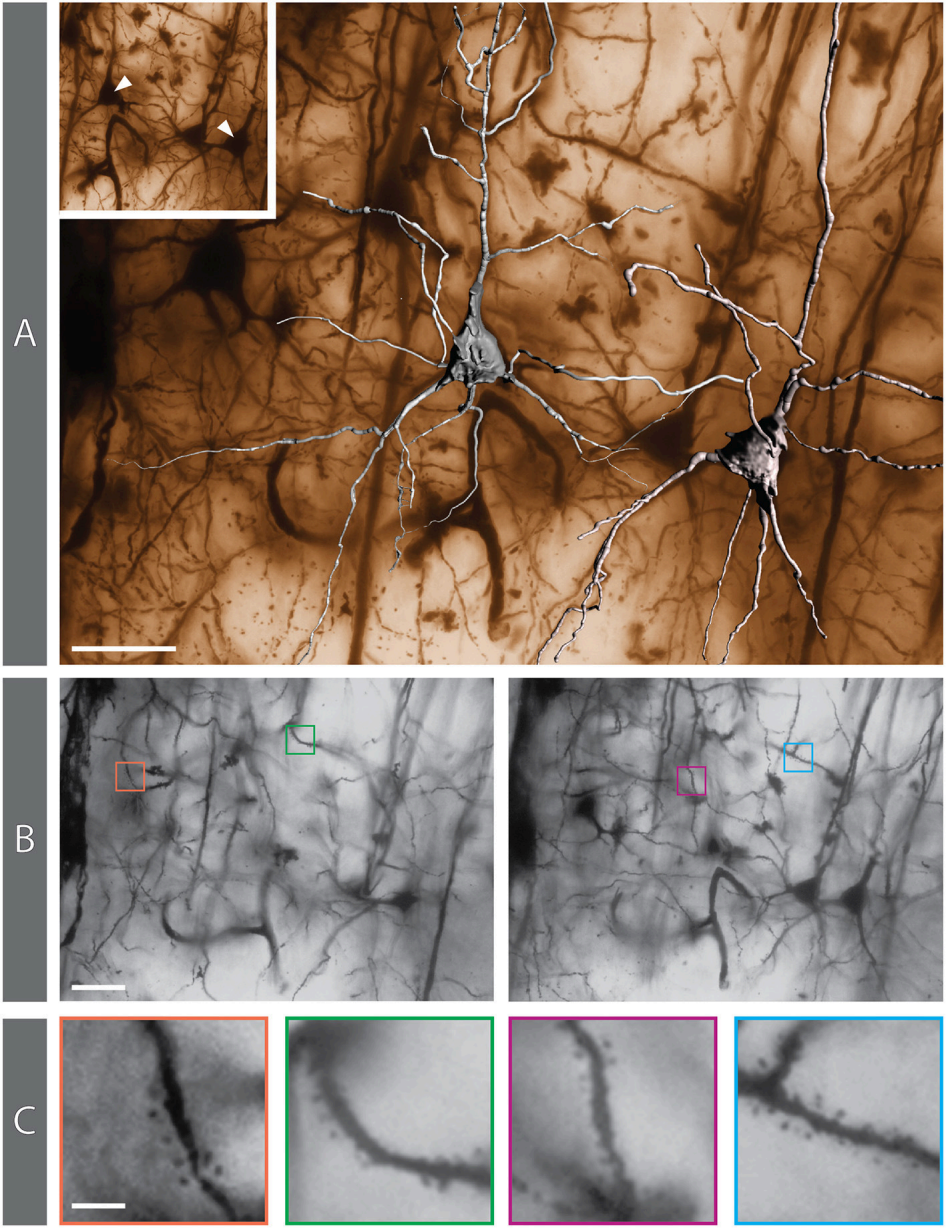
Golgi-impregnated preparation from the rabbit cerebral cortex. **(A)** 3D reconstruction of 2 pyramidal neurons superimposed on the matching minimum-intensity-projection (MIP) rendering obtained from a 250-image z-stack. The MIP algorithm searches, for each X-Y position in the stack, the darkest pixel along the z axis (the focusing plane) and assigns its value to the corresponding pixel in the final 2D image. The positions of the reconstructed neurons are indicated in the inset. **(B)** Two individual images from the z-stack (#27 and #58, respectively) in which dendritic spines can be appreciated (colored boxes). **(C)** The same 4 areas containing spines, shown at higher magnification. Scale bars: **(A,B)**, 30 μm; **(C)**, 5 μm.

The heterogeneity in the shape, size, extent, and orientation of dendritic arborizations was probably for Golgi a very striking finding that he depicted with care in his drawings (Figures 5–7). The fully stained dendritic tree and the complexity of dendritic arborizations are the real wonder revealed by Golgi impregnation. Distal dendritic branches can be followed in different focal planes (Supplementary Movie 1). The full staining of processes in Golgi's slides is demonstrated by the 3D reconstruction of pyramidal neurons (Figure 8A).

We have been able to identify dendritic spines though their visualization has required careful focusing (Supplementary Movie 1). They are especially evident in dendrites of some of the impregnated cortical pyramidal neurons (Figures 8B,C), while the dendrites of other pyramidal neurons appear smooth. Dendritic spines are difficult to identify in hippocampal neurons (e.g., Figure 5C).

A thin axon arising from the cell body could be seen in several impregnated neurons, especially at its origin from the cell body (Figure 8A; Supplementary Movie 1). The axon could be followed only for a short distance after his emergence, and axonal bifurcations were observed (Figure 8A).

**Figure 9:**
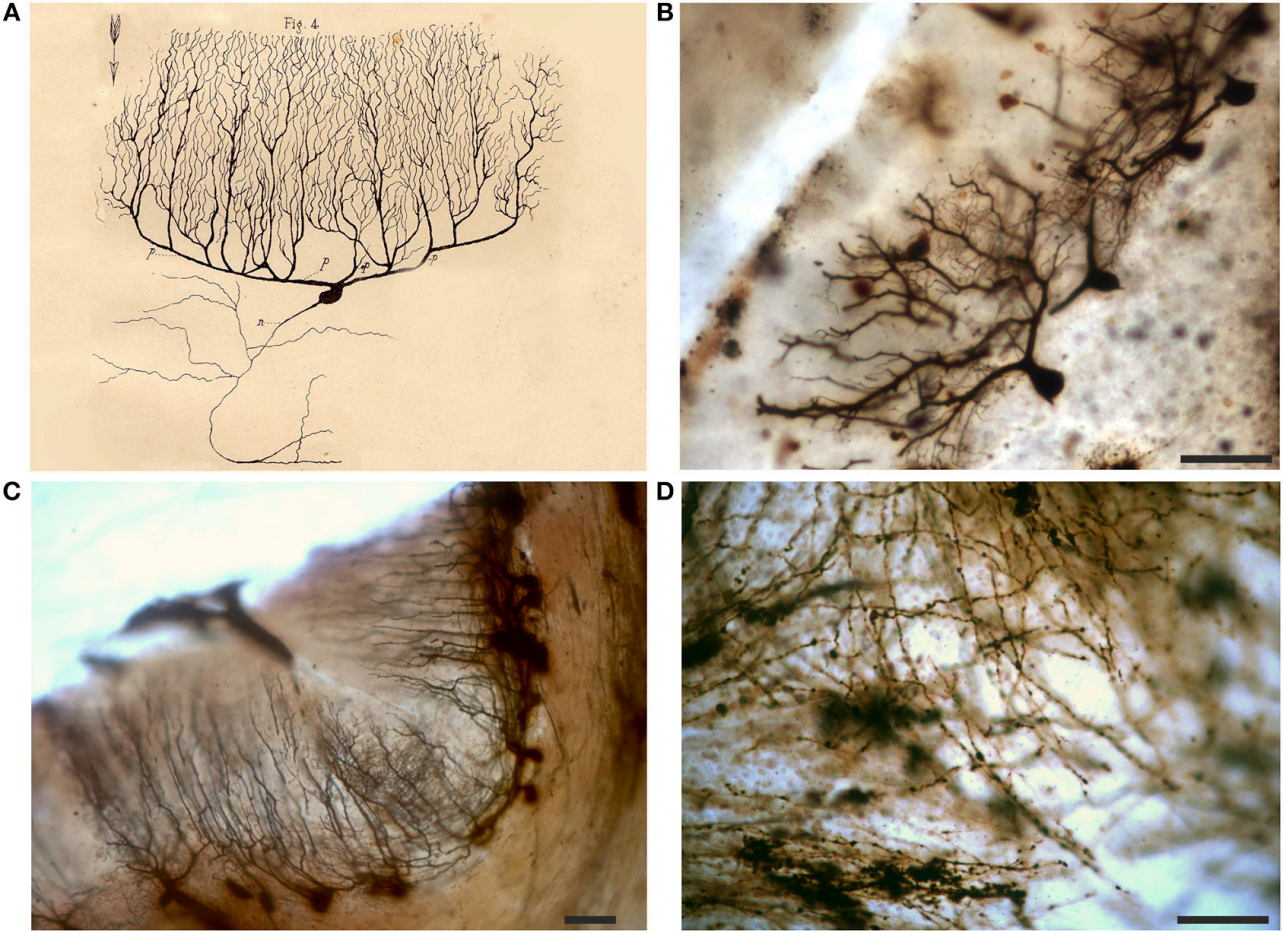
Drawing **(A)** and images **(B,C)** of Purkinje cells and white matter **(D)** from a Golgi-impregnated section of the cerebellum. **(A)** The drawing is from an essay on the structure of the nervous system (Golgi, 1883); p refers to “protoplasmic prolongations” (dendrites) and n to the “nervous prolongation”(axon); note the arrow which denotes the flow of the nervous impulse from the dendrites to the axon (Golgi inserted similar arrows in all his other drawings of neurons in this publication). Note in **(D)** the meshwork of varicose fibers running in different directions. Scale bars in **(B–D)**: 100 μm.

Slides of the cerebellar cortex mostly contain tissue fragments with many precipitates. In some fields, however, impregnated Purkinje cells can be found (Figures 9B,C), and spines are also visible in their dendritic tree (Figure 9B). The dendritic arbor of Purkinje cells should have impressed Golgi who drew them carefully. He seems also to have indicated with an arrow the direction of the nervous impulse from the Purkinje cell dendrites toward the cell body and axon (Figure 9A).

In the white matter of cerebellar folia, the axons, running in bundles with a parallel trajectory, are heavily impregnated in some slides. They show many varicosities and can be seen to converge into a central meshwork of fibers, running in different directions (Figure 9D).

### Cajal Stain

The label of two slides of the rabbit cerebral cortex specifies parietal and occipital cortex, respectively, and “Cajal” (Figure 3). Cajal developed two techniques to study intraneuronal organelles (DeFelipe and Jones, 1992; Garcia-Lopez et al., 2010; Merchan et al., 2016): reduced silver impregnation for the study of neurofibrils (Cajal, 1903), and gold chloride-sublimate impregnation for the study of astrocytes (Cajal, 1913). The slides were likely prepared in Golgi's laboratory in the early Twentieth century.

**Figure 10:**
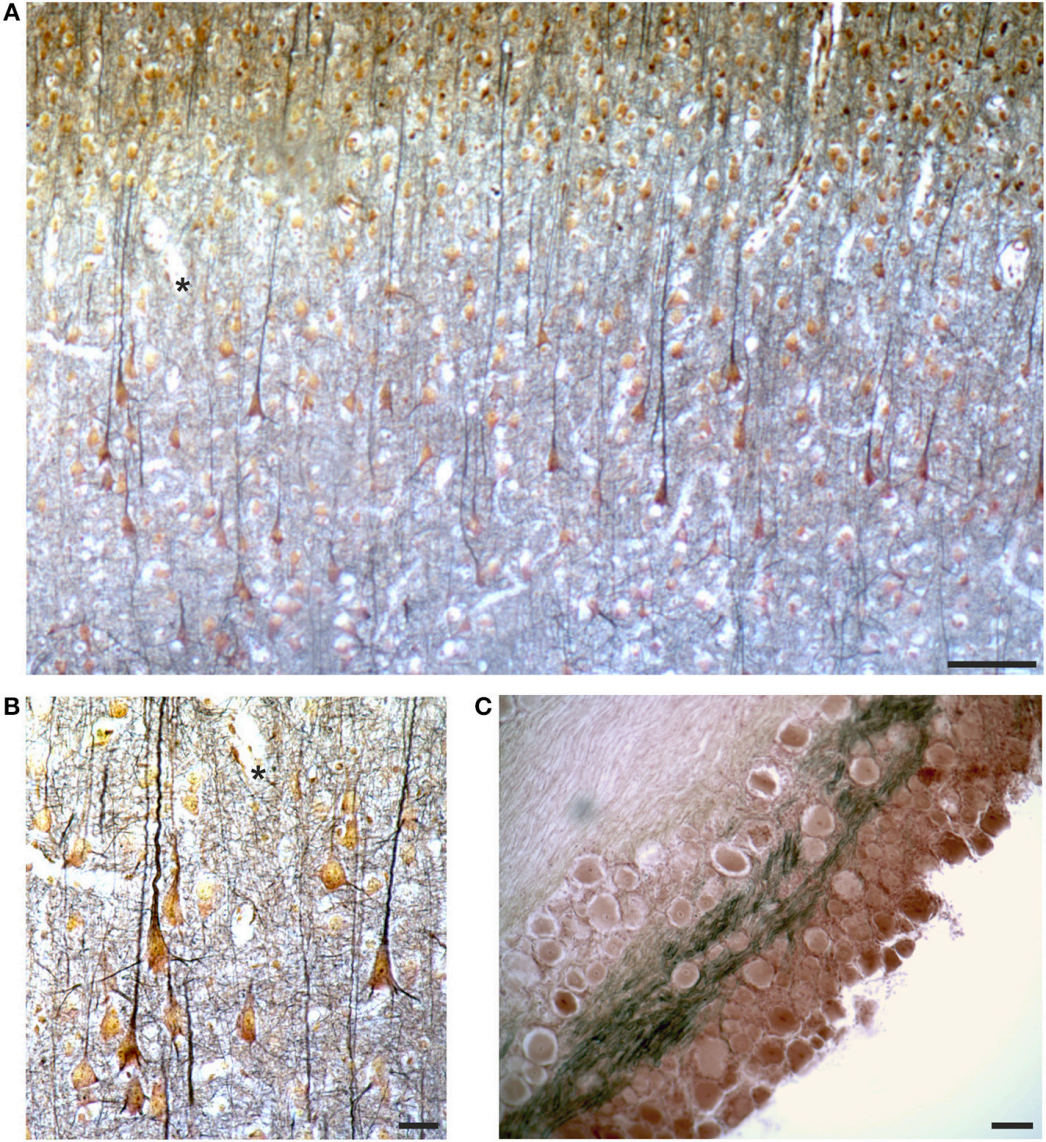
Images from the slides labeled as “Cajal” by Golgi (see Figure 3D), containing a section from the occipital cortex of the rabbit **(A,B)** and a ganglion **(C)**, respectively. In **(A,B)** the asterisks mark the same field for spatial reference. See text for comments on the Cajal stain. Scale bars: 100 μm in **(A,C)**, 25 μm in **(B)**.

These slides are coverslipped (Figure 3D) and the sections are relatively thin (do not require focal plane adjustments) and well-preserved. They contain gold-stained cell bodies, and the processes, especially the apical dendrite and some basal dendrites of pyramidal cells, are clearly delineated in black (Figures 10A,B), impregnating cytoskeletal elements (neurofibrils). The section of a dorsal root ganglion is also impregnated with the Cajal stain (Figure 10C). The sections seem impregnated with Cajal reduced silver nitrate, probably modified by Golgi, since modifications of the staining recipes were very common at that time.

## Discussion and Comments

The observation of Golgi's slides is certainly inspiring, though the microscopic examination of sections impregnated with the “black reaction” is not easy, especially due to their thickness. Golgi's sections seem to be thicker than those used by Cajal: according to DeFelipe and Jones (1992), Cajal used a thickness approaching 100 μm for Golgi-impregnated sections, whereas the thickness of Golgi's sections we examined is about twice as much. Similarly to Golgi, Cajal utilized a microtome to cut Golgi-impregnated sections and covered them with dammar resin without coverslipping; he used a “modern” Zeiss microscope and based his illustrations on camera lucida but also on freehand drawing (DeFelipe and Jones, 1992).

Golgi's methodological notes indicate that he worked out an ingenious strategy to examine the slides from both sides and devised a protocol to preserve adequately the sections for a long time. He therefore used to observe his material repeatedly over the years.

As for Golgi's findings, there is no doubt about the fact that he first saw neurons in their entirety and depicted for the first time a striking neuronal heterogeneity in different brain regions (olfactory bulb, neocortex, hippocampus, cerebellum). These observations, made from 1873 to 1885, paved the way to a topographical neuroanatomical description and graphic representation of central nervous system structures. Extremely important were his neurocytological discoveries, discussed below and documented by his histological preparations and drawings. These provided the bricks for further architectural and functional interpretations.

### Dendrites and Dendritic Spines

Golgi must have been certain to have followed-up dendrites until their endings with careful focusing, so that dendrites always end freely in his drawings. This contradicted Gerlach's theory of a dendritic network with fused arborizations, as Golgi firmly stated in his Nobel lecture in 1906 (Golgi, 1967). He ascribed to dendrites a trophic function, on the basis of observations of a proximity of dendrites to blood vessels (Golgi, 1885, 1967; Raviola and Mazzarello, 2011).

Golgi (1885) did not describe dendritic spines in his “fine anatomy” and acknowledged much later, in drawings of Purkinje cells in 1901, their existence though without any comment (DeFelipe, 2015). We here show that dendritic spines can be seen in his preparations. Cajal described (and named) spines in his earliest observations, in 1888, based on the Golgi impregnation of the avian cerebellum (DeFelipe, 2015).

Golgi (1967) stated in his Nobel lecture that he was not certain that the small dendritic protrusions represented distinctive features of dendrites since he had observed similar protrusions in glial cells and in the axon (probably axon varicosities). A likely hypothesis for Golgi's missed description of dendritic spines is that he believed that they could be artifacts. Cajal made “controls” of potential artifacts, checking the distribution of spines in different neuronal compartments (their presence in dendrites and absence in cell bodies), verifying their existence also by using methylene blue staining (García-López et al., 2007).

In his pioneering observations, Golgi should have been extremely worried about artifacts, and he saw lots of artifacts (precipitates) in his slides. His close friend Bizzozero had probably repeatedly warned Golgi that artifacts were the main drawback of histological stains. Even many years later, when Golgi's student Adelchi Negri showed him at the beginning of the Twentieth century the intraneuronal inclusions in the hippocampus of rabid dogs (the Negri bodies, which then became pathognomonic of rabies), Negri had to cut and stain (with the Mann staining) many dog brains to control that the inclusions were not staining artifacts (Kristensson et al., 1996; Mazzarello, 2010).

It should also be considered that the Golgi impregnation is especially effective in brain tissue from young animals, in which dendritic spines are especially evident (e.g., Rosoklija et al., 2003), as Cajal emphasized (Garcia-Lopez et al., 2010; Merchan et al., 2016). However, the animal's age (or the age at death for human autoptic specimens) was not specified for every sample in Golgi's “fine anatomy,” although Golgi (Golgi, 1885) mentioned to have used material from newborn animals and even fetuses for his preparations of the spinal cord.

### Axon and Axonal Branching

From his slides and drawings, it is obvious that Golgi could identify the axon at its emergence from neuronal cell bodies. He carefully examined the features of this thin single process and stated that it was different from dendrites and that its presence differentiates nerve cells from glial cells (“connective cells”) (Golgi, 1885).

Golgi thus discovered that the axon is a constant feature of neurons, and originates only from the cell body, and not also from dendrites as previously proposed. Golgi was reluctant to get involved in theoretical elaborations: he ironically defined his own attitude as “hypothesisphobia” (Golgi, 1903), and highly appreciated Tacitus' aphorism “I have never felt regret for having been silent; only for having spoken” (Mazzarello, 2010). However, he affirmed with vigor that the functional activity of nerve cells is effected by the single nerve prolongation via connections with their distant targets (Golgi, 1885), a concept that is a pillar of neuroscience.

In all his drawings of neurons that illustrate a treatise of the structure of the nervous system which has never been translated (Golgi, 1883), an arrow points from the dendrites to the axon (see the example in Figure 9A). This indicates that Golgi had understood the directionality of the nervous signal from dendrites to the “single nerve prolongation,” while in the preceding models of Gerlach and others this function was mainly assigned to the dendrites. Besides the hypothesis of a trophic function of dendrites mentioned above, in his Nobel lecture Golgi (1967) stated that the dendrites, which “are a direct product of the body of the nerve cell whose structure they reproduce…,” may share the specific function of this cell.

Golgi could follow separate axons running in bundles. However, he likely would have then lost the nerve fiber individuality at the convergence of different bundles, where intersection of fibers (and their overlap in different focal planes) can be seen at the microscopic observation of his preparations. Again, the section thickness, useful for the appreciation of neurons in their entirety, might have rendered difficult to decipher the fate of individual axons.

Furthermore, Golgi was able to identify axon collaterals, while the axon was believed to be unbranched at his times. This was a fundamental observation though it contributed to the overall idea of the nervous tissue composed of a “diffuse net.”

On the basis of axonal bifurcations, Golgi distinguished two types of multipolar nerve cells: type I cells with long axons giving off few collateral branches and maintaining its individuality; type II cells, with an axon dividing repeatedly shortly after its emergence from the cell body (see Figure 7A). This distinction is still valid nowadays.

His observations must have led Golgi to adhere to the concept of nervous net, a concept supported by his belief that the activity of the nervous system was due “not to the isolated action of individual cells but to the simultaneous activity of large groups of cells (Golgi, 1885, 1903).” In other words the different parts of the brain and spinal cord were viewed by Golgi almost as *functional fields* which could explain the complexity of brain activity (Mazzarello, 2018).

#### Final Remarks

It is difficult to examine Golgi's slides thinking of observations blind of knowledge accumulated in the following 150 years. However, looking at Golgi's slides it is unavoidable to guess the surprise of being able to observe nerve cells in their entirety for the first time and the effort of making sense of their intricacy.

## Author Contributions

MB, TC, SF, CT, SM, and GB examined the slides and took images. TC, CT, and GB analyzed the data for 3D reconstructions. TC and CT prepared the figures. AB and PM provided the slides and historical material. MB and PM wrote the manuscript and all authors contributed to the manuscript.

### Conflict of Interest Statement

The authors declare that the research was conducted in the absence of any commercial or financial relationships that could be construed as a potential conflict of interest.

## Supplementary Material

Supplementary Movie 1 This animation shows a representative field of view from the cerebral cortex of the rabbit brain, impregnated by the Golgi method. At the chosen X-Y location, 250 still images were acquired with a digital camera (see Methods), focusing the 40X objective at 0.3 μm steps along the z axis. Image acquisition started approximately 30–40 μm below the surface of the tissue and ended approximately 30–40 μm above the surface of the glass slide. Images were equalized in order to optimize contrast and combined into a “movie” that reproduces the experience of changing the focal plane while observing a Golgi preparation at the microscope. The animation is temporarily paused to highlight structures of interest.
